# Resolutions of Respect to the Memory of Dr. Willis F. Westmoreland

**Published:** 1890-08

**Authors:** 


### RESOLUTIONS OF RESPECT TO THE MEMORY OF DR. WILLIS F. WESTMORELAND.

The undersigned committee, appointed at a large meeting of the physicians of the city to prepare resolutions upon the death of Dr. Willis F. Westmoreland, in obedience to the expressed wishes of that meeting, respectfully submit the following tribute:

*Resolved*, That while we, the colleagues and co-workers of Willis F. Westmoreland, M. D., reverently accept the divine decree that takes from us our friend and brother, yet, as we consign to the tomb his familiar form, we would derive from his life of useful endeavor, of consecrated labor and of brilliant achievement, inspiring and profitable lessons.

To an intellect of remarkable natural endowment, he added the gathered treasures of a close and faithful student of books, of nature, and of men. To habits of industrious application, he brought methods of logical thought and of accurate investigation. The soundness of his judgment was proverbial. As a diagnostician he was pre-eminently skillful. His genial and unaffected manners, and his incisive, direct and logical reasoning constituted, at once, the explanation of his popularity and of his power.

As a surgeon, he stood among the foremost of the land, and modest in assertion, he was without a superior within the realm of this great art. As a citizen, a friend, a counsellor, thousands attest his nobility and his worth.

As a teacher, he was acknowledged the peer of the best, and his precepts, delivered within the hall of medical learning, will ring along the corridors of time until time shall be no more.

Though dead, he yet speaketh.

*Resolved*, That by his death the medical profession of this city —the arena of his active labors—the State, the country, has been deprived of an able member, a useful, patriotic and distinguished citizen, and while we deeply deplore his loss, we cherish the memory of his noble traits and emulate his praiseworthy example in the honorable lines of his unselfish life.

*Resolved*, That our sympathy be tendered the members of his family in their supreme sorrow, and that a copy of these resolutions be furnished the city papers and the medical press for publication.

WILLIAM S. ARMSTRONG, M. D.
H. V. M. MILLER, M. D.
JAMES B. BAIRD, M. D.
WILLIAM PERRIN NICOLSON, M. D.
C. F. BENSON, M. D.
HENRY BAK, M. D.
JAMES F. ALEXANDER, M. D.
FRANK H. ORME, M. D.

